# Type II caesarean scar pregnancy management by ultrasound-guided local lauromacrogol injection in combination with suction curettage: A case report

**DOI:** 10.1097/MD.0000000000019743

**Published:** 2020-04-24

**Authors:** Shuang-shuang Wei, Ding-heng Li, Zhi-fen Zhang, Wen-chao Sun, Cen-lin Jia

**Affiliations:** a Department of Gynecology, Hangzhou Women's Hospital, Hangzhou, China.; b Center of Reproductive Medicine.

**Keywords:** caesarean scar pregnancy, lauromacrogol, suction curettage

## Abstract

**Introduction:**

The implantation of a gestational sac within the scar of a previous caesarean delivery is defined as caesarean scar pregnancy (CSP), which is classified into two types: CSP I and CSP II. CSP II is life threatening, and no clear consensus for CSP II management exists.

**Patient concerns:**

A 31-year-old woman, gravida 1, para 1, with a previous caesarean delivery due to macrosomia, presented with an estimated 45 days of amenorrhea. The patient presented to the emergency department with vaginal bleeding for 1 day and no abdominal pain.

**Diagnoses:**

An ultrasound examination was performed demonstrating a viable fetus that was embedded in the caesarean scar area and was bulging through the wall of the uterus into the bladder without contact with the uterine cavity or cervical canal. A diagnosis of type II caesarean scar pregnancy was made.

**Interventions:**

Local lauromacrogol was used to reduce the gestational sac blood supply. Suction curettage was performed under the guidance of abdominal ultrasound 24 h later, and the amount of bleeding was 20 mL. The response to the treatment was monitored by serial beta-human chorionic gonadotropin (β-hCG).

**Outcomes:**

Patient was followed up with β-hCG weekly levels which became <10 mIU/mL after 4 weeks of treatment.

**Conclusion:**

Ultrasound-guided local lauromacrogol injection combined with suction curettage may be a safer and novel therapeutic method.

## Abstract 2

Supplemental Digital Content is available in the text

## 1 Introduction

The caesarean scar pregnancy (CSP) is a rare ectopic pregnancy involving a gestational sac that has implanted onto a previous caesarean scar.^[1]^ The incidence of CSP ranges from 1/1800 to 1/2200 pregnancies,^[2]^ and CSP has also continuously increased due to the increased rate of caesarean sections. Vial et al^[3]^ defined two types of CSP. CSP I refers to a gestational sac embedded in the scar that grows toward the uterine cavity. CSP II refers to a gestational sac implanted in the scar that progresses toward the myometrium and the uterine serosal layer. Patients with CSP II face a high risk of uterine rupture and vaginal bleeding, which can be life threatening due to decreased thickness of the uterine myometrium between the gestational sac and the bladder, especially when the thickness is <3 mm.^[4]^ Thus, early diagnosis and termination of the pregnancy in the first trimester is warranted. Several treatment options have been reported, including a medical approach, a surgical approach, and a combination of both. However, to date, the optimal management of CSP, especially CSP II, remains to be determined. In this study, ultrasound-guided local lauromacrogol injection combined with suction curettage was successful as a novel treatment in the management of a case of CSP II.

## 2 Case report

**Figure 1:**
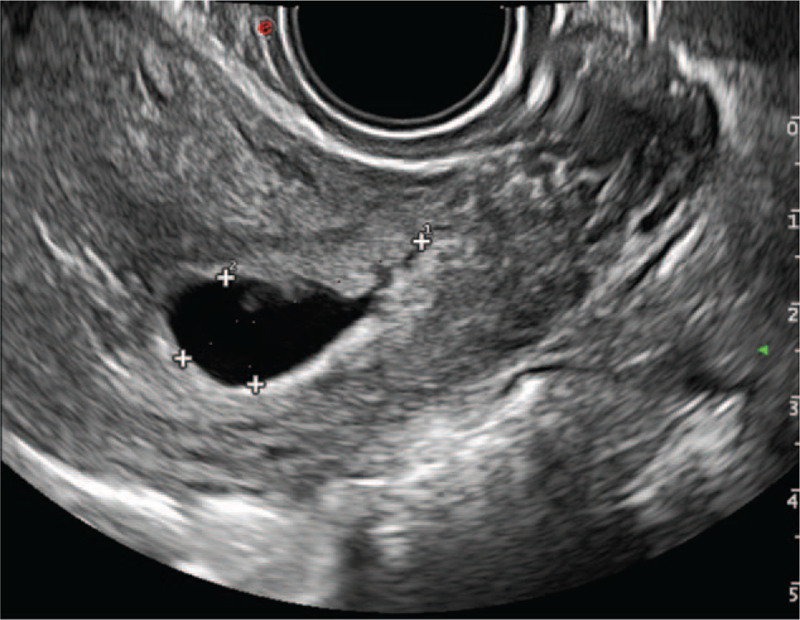
Colour power Doppler image showing the caesarean scar pregnancy in the lower uterine segment.

The patient was a 31-year-old woman, gravida 1, with a previous caesarean delivery due to macrosomia, with an estimated 45 days of amenorrhea. The patient presented to the emergency department with vaginal bleeding for 1 day and no abdominal pain. Her past medical history was unremarkable. We confirmed CSP II using the following transvaginal ultrasonographic parameters:

1. a the gestational sac size of 2.1 × 1.8 × 1.0 cm,
2. a viable fetus that was embedded in the caesarean scar area and was bulging through the wall of the uterus into the bladder without contact with the uterine cavity or cervical canal,
3. a myometrial thickness between the bladder and gestational sac of 0.23 cm, and
4. a rich vascular image in the area of the caesarean scar (Fig. 1).

The transvaginal ultrasound also showed that the ovaries appeared normal, and the pouch of Douglas had no fluid. The patient's β-hCG serum level was 25,154 mIU/mL. Because she was hemodynamically stable, the patient chose local lauromacrogol injection combined with aspiration.

**Figure 2:**
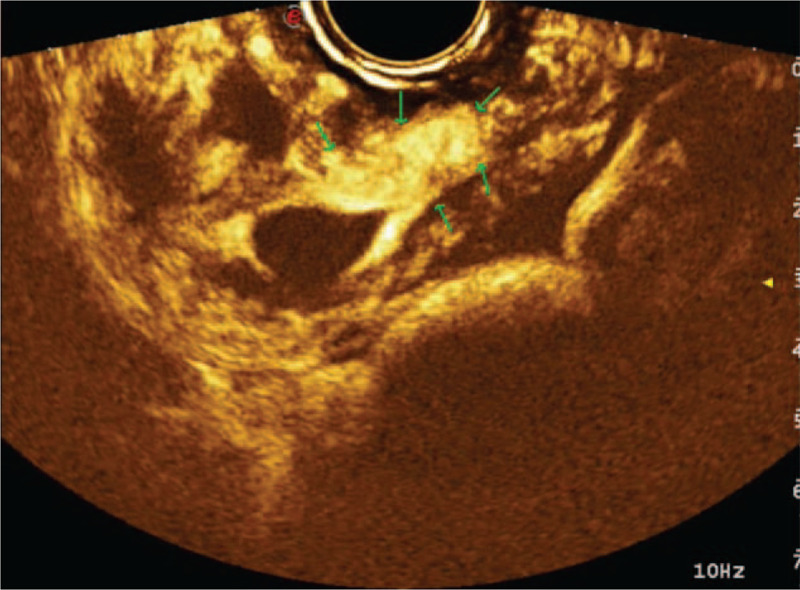
Shows that the incision range of villus implantation, and the blood perfusion in the villus implantation area is rich.

The patient fasted for 2 h before surgery. Five milliliters of sulfur hexafluoride microbubble contrast agent was intravenously injected. The location and size of the gestational sac, the myometrium between the bladder and gestational sac, and the blood supply of the surrounding area were observed under contrast-enhanced ultrasound (Fig. 2). A 21-gauge needle was used to puncture into the uterine cavity under the guidance of vaginal ultrasound. Fifteen milliliters of lauromacrogol was slowly injected at multiple points around the gestational sac until the ring of the pregnancy sac was strengthened like a “donut,” which indicated very little blood flow (Video 1). Then, the pregnancy sac was punctured again to extract the sac fluid. After sclerotherapy, vaginal ultrasonography was performed to examine the blood supply to the peripheral tissue of the pregnancy sac. Suction curettage was performed under the guidance of abdominal ultrasound 24 h later, and the amount of bleeding was 20 mL. Specimens were sent for pathological examination. The β-hCG level was 3736 after 1 day of suction curettage. The levels of β-HCG were determined weekly. The β-hCG level was 364.5 mIU/mL after 1 week of suction curettage and became <10 mIU/mL after 4 weeks of treatment. The pathological reports suggest that the sample for inspection was villous tissue. Her menstruation recovered after 23 days when the serum β-hCG level returned to normal. Patient has provided informed consent for publication of the case.

## 3 Discussion

CSP I is more conducive to maintaining pregnancy, and CSP II is more prone to severe hemorrhage and uterine rupture.^[5]^ Early diagnosis of CSP II is essential to avoid complications. However, the diagnosis of CSP may be delayed because it is difficult to differentiate CSP from an abortion in progress or a cervical pregnancy. Therefore, early suspicion of CSP using ultrasonography might be helpful toward confirming a diagnosis and classifying the CSP type.^[6,7]^ In this case, the myometrial layer between the bladder and sac was thin, the gestational sac grew toward the bladder and abdominal cavity, and abundant vascularization was present within the CSP site. We confirmed that the CSP type in this case is CSP II. These findings were also confirmed by MRI. This patient may face life-threatening uterine hemorrhage, uterine perforation, bladder injury, and persistence of trophoblasts. Current treatment options for CSP II include medical management, surgical management, or a combination of these methods. However, none of the current management strategies helps predict which therapy is more likely to be effective.^[5,6]^

In recent decades, suction curettage after uterine artery embolization (UEA) has been used as an effective treatment for CSP II to reduce the risk of severe hemorrhage.^[8,9]^ UEA can accurately detect and block blood flow in the uterine arteries to reduce the blood supply to the gestational sac, induce ischemic necrosis in the gestational sac, effectively terminate embryonic development, and rapidly deactivate trophoblasts, thus, efficiently reducing the bleeding risk during suction curettage.^[2]^ However, after blood flow in the uterine arteries is blocked by UEA, some patients may face problems, such as pain, nausea, vomiting, fever, the establishment of extensive collateral circulation, and even ovarian dysfunction.^[10,11]^ Moreover, UAE requires expensive digital imaging equipment. Lastly, the most serious complication of UEA is pulmonary embolism, which is rare but life threatening. Hence, the exploration of an effective new treatment for patients with CSP II is very important.

Lauromacrogol is widely used as a sclerosant for the treatment of various conditions, including gastrointestinal hemorrhage, sputum, and cystic disease.^[12,13]^ Because our patient has only one living child, we tried to use local lauromacrogol injection instead of UEA in this case to reduce the gestational sac blood supply. Lauromacrogol can seal off the veins located within the caesarean scar, which does not affect the blood supply of the ovary. Lauromacrogol exerts its sclerosant effects in 2 ways.^[10]^ First, direct intravascular lauromacrogol injection can wreck vascular endothelial cells in the veins around the injection site, promote local thrombosis, stimulate the surrounding tissue of ruptured blood vessels to form a protective layer of fibrous tissue and enhance vascular resistance, and cause regional vascular compression and hemostasis. This latter action can slow down blood flow and promote vascular protection to achieve hemostasis. Second, paravenous lauromacrogol injection mainly causes superficial small-area fibrosis in the veins around the injection site, causing vascular oppression and blockage. Because local lauromacrogol injection causes regional vessel oppression and hemostasis, ovarian damage is avoided.^[10]^ In this case, the amount of bleeding was 20 mL during suction curettage after lauromacrogol injection. The amount of bleeding in this method is similar to that in the method used by Jian Qiu in which hysteroscopy is performed after UEA.^[2]^ Therefore, local lauromacrogol injection can be used as an effective treatment for CSP II to reduce the amount of bleeding. Furthermore, the patient's reproductive function was preserved, and her menstrual cycles returned quickly.^[10,14]^ Lastly, lauromacrogol may serve to relieve pain.^[10,15]^

If the patient presents with severe abdominal pain and heavy vaginal bleeding, there should be increased concern about an impending rupture. The most important early clinical feature of CSP is vaginal bleeding due to a short interval between the current and previous pregnancies, especially if the interval is <1 year.^[16,17]^ In our study, the patient presented with vaginal bleeding without acute abdominal pain. The examination by transvaginal ultrasound showed that the thickness of the uterine myometrium between the gestational sac and the bladder wall was 0.23 cm. Therefore, this treatment is safe, and ultrasound-guided local lauromacrogol injection can provide protection when the gynecologist requires additional information for surgical intervention. However, for patients in whom the myometrium between the bladder and sac is absent, this therapy is not suitable due to difficulty with the injection. Hence, this novel treatment has limitations.

## Author contributions

**Methodology:** Ding-heng Li, Zhi-fen Zhang.

**Project administration:** Cen-lin Jia.

**Resources:** Shuang-shuang Wei, Ding-heng Li.

**Software:** Wen-chao Sun.

**Writing – original draft:** Shuang-shuang Wei.

**Writing – review & editing:** Shuang-shuang Wei.

Shuang-shuang Wei orcid: 0000-0002-9457-9628.

## Supplementary Material

Supplemental Digital Content

## References

[1] Ma Y Shao M Shao X Analysis of risk factors for intraoperative hemorrhage of cesarean scar pregnancy. Medicine (Baltimore) 2017;96:e7327. 28640149 10.1097/MD.0000000000007327 PMC5484259

[2] Qiu J Fu Y Xu J Analysis on clinical effects of dilation and curettage guided by ultrasonography versus hysteroscopy after uterine artery embolization in the treatment of cesarean scar pregnancy. Ther Clin Risk Manag 2019;15:83–9. 30662266 10.2147/TCRM.S184387 PMC6327891

[3] Vial Y Petignat P Hohlfeld P Pregnancy in a cesarean scar. Ultrasound Obstet Gyn 2000;16:592–3. 10.1046/j.1469-0705.2000.00300-2.x 11169360

[4] Sun YY Xi XW Yan Q Management of type II unruptured cesarean scar pregnancy: Comparison of gestational mass excision and uterine artery embolization combined with methotrexate. Taiwan J Obstet Gynecol 2015;54:489–92. 26522097 10.1016/j.tjog.2015.08.002

[5] Kim SY Yoon SR Kim MJ Cesarean scar pregnancy; diagnosis and management between 2003 and 2015 in a single center. Taiwan J Obstet Gynecol 2018;57:688–91. 30342652 10.1016/j.tjog.2018.08.013

[6] Zhang H Huang J Wu X Clinical classification and treatment of cesarean scar pregnancy. J Obstet Gynaecol Res 2017;43:653–61. doi: 10.1111/jog.13267. 28150370 10.1111/jog.13267

[7] Boujoual M Moussaoui DR Cesarean scar pregnancies. Pan Afr Med J 2018;30:228. 30574246 10.11604/pamj.2018.30.228.14384 PMC6295290

[8] Chen H Zhou J Wang H The treatment of cesarean scar pregnancy with uterine artery embolization and curettage as compared to transvaginal hysterotomy. Eur J Obstet Gynecol Reprod Biol 2017;214:44–9. 28472704 10.1016/j.ejogrb.2017.04.032

[9] Qiao B Zhang Z Li Y Uterine artery embolization versus methotrexate for cesarean scar pregnancy in a Chinese population: a meta-analysis. J Minim Invasive Gynecol 2016;23:1040–8. 27553186 10.1016/j.jmig.2016.08.819

[10] Chai ZY Yu L Liu MM Evaluation of the efficacy of ultrasound-guided local lauromacrogol injection combined with aspiration for cesarean scar pregnancy: a novel treatment. Gynecol Obstet Invest 2018;83:306–12. 29208846 10.1159/000485099

[11] Arthur R Kachura J Liu G Laparoscopic myomectomy versus uterine artery embolization: long-term impact on markers of ovarian reserve. J Obstet Gynaecol Can 2014;36:240–7. 24612893 10.1016/S1701-2163(15)30632-0

[12] Ramadani A Jovanovska RP Trajkovska M Comparison of argon plasma coagulation and injection therapy with adrenalin and polidocanolin the management of bleeding angiodysplasia in upper gastrointestinal tract. Pril (Makedon Akad Nauk Umet Odd Med Nauki) 2018;39:63–8. 30864370 10.2478/prilozi-2018-0043

[13] Gupta G Pandit RS Jerath N Severe life-threatening hypersensitivity reaction to polidocanol in a case of recurrent aneurysmal bone cyst. J Clin Orthop Trauma 2019;10:414–7. 30828217 10.1016/j.jcot.2018.05.010 PMC6383131

[14] Giampaolino P De Rosa N Morra I Bifulco management of cesarean scar pregnancy: a single-institution retrospective review. Biomed Res Int 2018;2018: doi: 10.1155/2018/6486407. 10.1155/2018/6486407 PMC5859871 29693012

[15] Gao L Huang Z Gao J Uterine artery embolization followed by dilation and curettage within 24 hours compared with systemic methotrexate for cesarean scar pregnancy. Int J Gynaecol Obstet 2014;127:147–51. 25212971 10.1016/j.ijgo.2014.05.005

[16] Luo L Ruan X Li C Early clinical features and risk factors for cesarean scar pregnancy: a retrospective case-control study. Gynecol Endocrinol 2018;1–5. 30430877 10.1080/09513590.2018.1526276

[17] Canelas CM Shih RD Clayton LM Repeat acute abdomen and hemoperitoneum during the same pregnancy due to a rupturedectopic treated by salpingostomy. Am J Emerg Med 2017;35:942.e1–3. 10.1016/j.ajem.2017.01.024 28104324

